# Facet overhang: A novel parameter in the pathophysiology of multifidus muscle atrophy

**DOI:** 10.1002/ca.23923

**Published:** 2022-06-23

**Authors:** Michelle Chua, Khalil Salame, Morsi Khashan, Dror Ofir, Uri Hochberg, Ran Ankory, Zvi Lidar, Gilad J. Regev

**Affiliations:** ^1^ Department of Neurosurgery Tel‐Aviv Sourasky Medical Center Tel‐Aviv; ^2^ Sackler Faculty of Medicine Tel Aviv University Tel‐Aviv

**Keywords:** cross‐sectional area, facet arthropathy, fatty infiltration, lumbar spinal stenosis, multifidus, muscle atrophy

## Abstract

The relationship between degenerative zygapophysial joint (facet) arthropathy and multifidus muscle atrophy has not been rigorously evaluated. The purpose of this study was to determine if specific morphological features of degenerative facet arthropathy are correlated with multifidus muscle atrophy. We retrospectively reviewed medical records and imaging studies of patients with lumbar spinal stenosis. Facet overhang, bridging osteophyte formation, facet effusion, and facet angles were evaluated by univariable and multivariable regression to identify independent associations with deep and superficial parts of the multifidus total cross‐sectional area (tCSA), functional cross‐sectional area (fnCSA), and fatty infiltration (FI). Facet overhang was classified as severe in 50 females (53.2%) versus 56 males (36.9%) (*p* = 0.030). Severity of facet overhang and female sex were independently associated with smaller deep part of the multifidus tCSA and fnCSA as well as higher FI, reflecting greater atrophy of the deep region compared to total muscle mass. In comparison, severe facet overhang (*p* < 0.001; OR = 3.47, 95% CI = 2.13–5.66) and female sex (*p* < 0.001; OR = 4.19, 95% CI = 2.58–6.79) were independently associated only with higher superficial part of the multifidus FI, reflecting muscle steatosis without significant lean muscle atrophy. In patients with degenerative lumbar spinal stenosis, facet overhang is an independent risk factor for deep part of the multifidus atrophy. Bridging osteophyte formation, facet effusion, and facet angles were not independently associated with deep part of the multifidus atrophy.

## 1 INTRODUCTION

The multifidus muscle is an important stabilizer of the lumbar spine (MacDonald et al., 2006; Macintosh et al., 1986; Regev et al., 2010; Ward et al., 2009). In previous studies, multifidus atrophy has been associated with chronic low back pain, higher functional disability in patients with degenerative spinal pathology, and poorer clinical outcomes following surgical treatment of lumbar degenerative diseases (Cooley et al., 2018; Fortin et al., 2017; Jermy et al., 2020; Wang et al., 2020).

Lumbar spinal stenosis is usually caused by a series of degenerative changes in the intervertebral disc and corresponding zygapophysial (facet) joints. Previous studies have demonstrated a correlation between degenerative changes in the intervertebral disc and multifidus atrophy due to dysregulation of inflammatory pathways (James et al., 2018; Sun et al., 2017). However, the relationship between specific features of facet joint degeneration and development of multifidus atrophy has not been extensively studied.

Intervertebral disc degeneration can lead to reduction in disc height and, occasionally, development of spondylolisthesis or scoliosis due to segmental hypermobility (Sengupta & Herkowitz, 2005). These structural alterations further influence the facet joints leading to characteristic anatomical changes. Typically, anterior subluxation of the inferior articular process may lead to posterior overhang of the facet joint, which is increased due to the formation of posterior bridging osteophytes. Additionally, subluxation of the facet joint can lead to opening of the joint space.

The multifidus muscle is innervated by the medial branch of the dorsal ramus, which courses around the lateral border of the superior articular process before descending medially along the lamina (Regev et al., 2009; Shuang et al., 2015). The deep part of the multifidus fibers arise from the superior articular process and lamina and insert superiorly onto the facet capsule and lamina whereas the superficial part of the multifidus fibers originate from the mammillary process inferiorly and insert onto the spinous process and supraspinous ligament (Lonnemann et al., 2008). Evidence from functional studies suggest that deep fibers of the multifidus muscle possess a primarily tonic stabilizing function whereas superficial fibers of the multifidus muscle possess a phasic function, acting as extensors and rotators of the spine similar to the erector spinae muscles (Donisch & Basmajian, 1972; Moseley et al., 2002; Regev et al., 2010).

In this study, we evaluated the severity of facet joint degeneration and multifidus muscle atrophy in patients with lumbar spinal stenosis. Specific morphological features of facet joint arthropathy were then correlated with muscle atrophy of the deep or superficial part of the multifidus.

## 2 MATERIALS AND METHODS

Medical records and imaging studies of patients with clinically and radiologically defined lumbar spinal stenosis without prior lumbar surgery that were referred to our clinic for spinal decompression from 1 January 2016 to 31 December 2020 were retrospectively reviewed.

### 2.1 Evaluation of facet arthropathy

**FIGURE 1:**
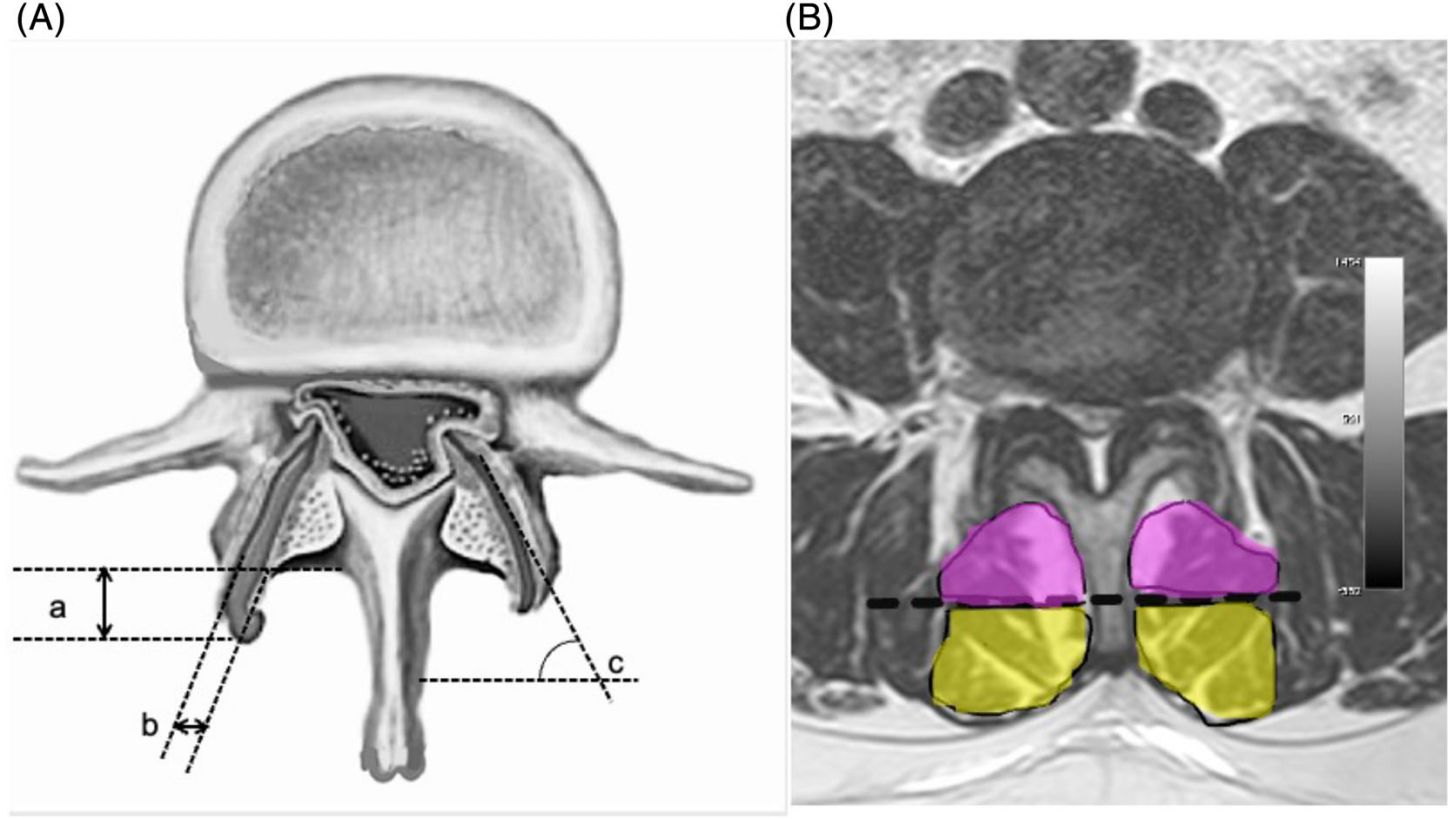
(A) Facet joint morphological features measurements: Facet overhang (a), facet joint opening (b), and facet angle (c). (B) Muscle measurements. Magnetic resonance image, T2 weighted axial cut showing the margins of the multifidus. (Deep fibers of the multifidus muscle were defined as short interlaminar fibers located deep to the midpoint between the base and tip of the spinous process. Deep part of the multifidus tCSA is highlighted in purple and superficial part tCSA is highlighted in yellow

Facet measurements were obtained bilaterally at the stenotic level, or levels in multilevel disease, on axial T2 weighted MR images. Facet overhang was defined as the distance measured from the base of the lamina to the posterior margin at the midlevel of the facet joint (Figure 1A). Mild, moderate, and severe facet overhangs were defined by an anteroposterior distance of <5 mm, 5–7 mm, and >7 mm, respectively (Ming & Sun, 2021). Bridging osteophytes were defined as heterotopic bone formation across the posterior aspect of the facet joint.

Facet joint opening was defined as a measurable, curvilinear, high intensity signal within the facet joint, which closely matched the signal intensity of cerebrospinal fluid (Figure 1A), and graded according a previously described classification system. Grade 0 was defined as absence of facet effusion, grade 1 was defined as measurable facet effusion <1.5 mm in width, and grade 2 was defined as large facet effusion ≥1.5 mm in width (Tamai et al., 2017).

Facet joint angle was measured as the angle subtended by the plane of the facet joint with respect to the horizontal plane (Figure 1A) and sagittal facet orientation was defined by a facet angle of >45°.

### 2.2 Multifidus muscle measurements

The deep part of the multifidus was defined as the region of the muscle located deep to the midpoint between the base and tip of the spinous process (Figure 1B). Deep and superficial parts of the multifidus total cross‐sectional area (tCSA), functional cross‐sectional area (fnCSA), and fatty infiltration (FI) were measured bilaterally at the midlevel of the facet joint at the stenotic spinal level. Multifidus fnCSA was measured using the thresholding technique as described by Fortin and Battie, (Fortin & Battie, 2012). Multifidus FI was defined as the ratio of multifidus tCSA minus multifidus fnCSA divided by multifidus tCSA.

### 2.3 Statistical analysis

Statistical analysis was performed using R version 3.6.3 (http://www.r-project.org). In univariable analysis, variables were compared between groups by Fisher's exact test for categorical variables and the Wilcoxon signed‐rank test or one‐way analysis of variance (ANOVA) for numerical variables. Statistical significance was defined as *p* < 0.05. Multivariable analysis was performed using multiple logistic regression. Parameters were included in multivariable analysis based on clinical significance and statistical significance in univariable analysis.

## 3 RESULTS

A total of 246 facet joints were evaluated in 107 patients with lumbar spinal stenosis. There were 64 males (59.8%) and 43 females (40.2%). Measurements revealed 106 facets (43.1%) with severe facet overhang, and 57 facets (23.2%) with large facet joint opening (Table 1). Severity of facet overhang was associated with bridging osteophyte formation (*p* < 0.001) and severity of facet effusion (*p* = 0.005) but not with facet orientation (*p* = 0.656).

**TABLE 1:** Patient demographics and musculoskeletal characteristics

	Patients
	(*N* = 107)
Age (years)	69.0 ± 10.1
Age < 70 years	53 (49.5%)
Age ≥ 70 years	54 (50.5%)
Male	64 (59.8%)
Female	43 (40.2%)
Single level lumbar spinal stenosis	91 (85.0%)
Multilevel lumbar spinal stenosis	16 (15.0%)

	Facet joints
	(*N* = 246)
Level of lumbar spinal stenosis	
L2/L3	24 (9.8%)
L3/L4	62 (25.2%)
L4/L5	154 (62.6%)
L5/S1	6 (2.4%)
Facet overhang (mm)	9.4 ± 5.4
Mild facet overhang	111 (45.1%)
Moderate facet overhang	29 (11.8%)
Severe facet overhang	106 (43.1%)
No bridging osteophyte	151 (61.4%)
Bridging osteophyte	95 (38.6%)
No facet effusion	111 (45.1%)
Small facet effusion	78 (31.7%)
Large facet effusion	57 (23.2%)
Facet angle (degrees)	56.2 ± 10.5
Coronal facet orientation	45 (18.3%)
Sagittal facet orientation	201 (81.7%)

In univariable analysis, severity of facet overhang was associated with bridging osteophyte formation (*p* < 0.001) and severity of facet effusion (*p* = 0.005) but not with facet orientation (*p* = 0.656). Level of spinal stenosis was associated with deep multifidus tCSA (*p* = 0.003), deep multifidus fnCSA (*p* < 0.001), deep multifidus FI (*p* < 0.0001), superficial multifidus tCSA (*p* < 0.0001), and superficial multifidus fnCSA (*p* < 0.0001). In addition, multilevel spinal stenosis was associated with deep multifidus tCSA (*p* = 0.002), deep multifidus fnCSA (*p* < 0.001), deep multifidus FI (*p* = 0.008), and superficial multifidus tCSA (*p* = 0.004).

### 3.1 Age and sex differences in facet arthropathy

Facet overhang was found to be mild in 33 females (35.1%) versus 78 males (51.3%) and severe in 50 females (53.2%) versus 56 males (36.9%) (*p* = 0.030). There were no other significant sex related differences and no significant age related differences in measures of facet arthropathy (Table 2).

**TABLE 2:** Univariable analysis for associations between patient characteristics and degenerative facet joint changes

	Female	Male	*p*	<70 years	≥70 years	*p*
	(*N* = 94)	(*N* = 152)		(*N* = 120)	(*N* = 126)	
Mild facet overhang	33 (35.1%)	78 (51.3%)		57 (47.5%)	54 (42.9%)	0.776
Moderate facet overhang	11 (11.7%)	18 (11.8%)	0.030	14 (11.7%)	15 (11.9%)	
Severe facet overhang	50 (53.2%)	56 (36.9%)		49 (40.8%)	57 (45.2%)	
No bridging osteophyte	57 (60.6%)	94 (61.8%)	0.893	73 (60.8%)	78 (61.9%)	0.896
Bridging osteophyte	37 (39.4%)	58 (38.2%)		47 (39.2%)	48 (38.1%)	
No facet effusion	38 (40.4%)	73 (48.1%)		57 (47.5%)	54 (42.9%)	0.646
Small facet effusion	29 (30.9%)	49 (32.2%)	0.252	38 (31.7%)	40 (31.7%)	
Large face effusion	27 (28.7%)	30 (19.7%)		25 (20.8%)	32 (25.4%)	
Coronal facet orientation	20 (21.3%)	25 (16.4%)	0.397	21 (17.5%)	24 (19.0%)	0.869
Sagittal facet orientation	74 (78.7%)	127 (83.6%)		99 (82.5%)	102 (81.0%)	

### 3.2 Association of facet arthropathy with multifidus atrophy

The superficial part of the multifidus fnCSA was not significantly associated with facet overhang, bridging osteophyte formation, facet effusion, or facet orientation (Table 3). In multivariable analysis, severe facet overhang (*p* < 0.0001; OR = 3.03; 95% CI = 1.64–5.62) and bridging osteophyte formation (*p* = 0.019, OR = 2.04, 95% CI = 1.13–3.69) were independently associated with larger superficial part of the multifidus tCSA whereas severe facet overhang (*p* < 0.001; OR = 3.47, 95% CI = 2.13–5.66) and female sex (*p* < 0.001; OR = 4.19, 95% CI = 2.58–6.79) were independently associated with higher superficial part of the multifidus FI, reflecting muscle steatosis without significant lean muscle atrophy.

**TABLE 3:** Univariable analysis for associations between degenerative facet joint changes and multifidus atrophy

	Deep multifidus tCSA (cm^2^)	*p*	Deep multifidus fnCSA (cm^2^)	*p*	Deep multifidus FI	*p*	Superficial multifidus tCSA (cm^2^)	*p*	Superficial multifidus fnCSA (cm^2^)	*p*	Superficial multifidus FI	*p*
Mild facet overhang	3.2 ± 0.8	<0.001	1.3 ± 0.6	<0.001	58.4 ± 18.2%	<0.001	5.1 ± 1.8	<0.001	3.2 ± 1.5	0.824	37.4 ± 15.0%	<0.001
Moderate facet overhang	2.6 ± 0.6		0.9 ± 0.5		65.3 ± 19.4%		5.6 ± 1.8		3.4 ± 1.7		42.1 ± 17.4%	
Severe facet overhang	2.4 ± 0.7		0.5 ± 0.4		80.8 ± 13.9%		6.7 ± 1.6		3.2 ± 1.4		51.3 ± 19.3%	
No bridging osteophyte	3.0 ± 0.9	0.824	1.1 ± 0.7	0.404	64.3 ± 19.7%	0.439	5.3 ± 1.7	<0.001	3.1 ± 1.4	0.096	41.5 ± 16.8%	0.022
Bridging osteophyte	2.5 ± 0.7		0.6 ± 0.5		76.1 ± 17.5%		6.6 ± 1.8		3.4 ± 1.6		47.8 ± 20.1%	
No facet effusion	2.8 ± 0.9	0.637	1.0 ± 0.7	0.035	65.8 ± 22.0%	0.006	5.7 ± 2.1	0.358	3.1 ± 1.5	0.461	44.6 ± 18.3%	0.326
Small facet effusion	2.7 ± 0.8		0.9 ± 0.6		68.1 ± 16.8%		5.8 ± 1.6		3.3 ± 1.4		41.5 ± 17.0%	
Large facet effusion	2.9 ± 0.8		0.7 ± 0.6		75.9 ± 17.0%		6.1 ± 1.7		3.3 ± 1.6		46.1 ± 20.2%	
Coronal facet orientation	3.3 ± 0.9	0.001	0.9 ± 0.7	0.802	72.2 ± 17.9%	0.263	6.0 ± 1.4	0.583	3.4 ± 1.4	0.202	42.7 ± 19.2%	0.490
Sagittal facet orientation	2.7 ± 0.8		0.9 ± 0.6		68.2 ± 20.0%		5.8 ± 2.0		3.2 ± 1.5		44.2 ± 18.3%	

In comparison, severity of facet overhang and female sex were, in multivariable analysis, independently associated with smaller deep part of the multifidus tCSA and fnCSA, and higher FI, reflecting greater atrophy of this muscle region compared to the total muscle mass (Table 4).

**TABLE 4:** Multivariable analysis

	Predictors	OR (95% CI)	*p*
Deep multifidus FI	**Severity of facet overhang**		
	Moderate facet overhang	2.35 (1.10–5.03)	0.028
	Severe facet overhang	8.69 (4.96–15.2)	<0.001
	**Male gender**	0.40 (0.25–0.64)	<0.001
	**Multilevel spinal stenosis**	1.32 (0.67–2.60)	0.425
	**Level of spinal stenosis**		
	Spinal stenosis at L3/L4	2.42 (1.01–5.80)	0.048
	Spinal stenosis at L4/L5	5.66 (2.35–13.7)	<0.001
	Spinal stenosis at L5/S1	9.35 (2.16–40.6)	0.003
Deep multifidus tCSA	**Severity of facet overhang**		
	Moderate facet overhang	0.18 (0.08–0.38)	<0.001
	Severe facet overhang	0.08 (0.05–0.15)	<0.001
	**Male gender**	2.26 (1.40–3.64)	<0.001
	**Multilevel spinal stenosis**	2.30 (1.14–4.64)	0.020
	**Level of spinal stenosis**		
	Spinal stenosis at L3/L4	3.27 (1.39–7.69)	0.007
	Spinal stenosis at L4/L5	15.3 (6.42–36.7)	<0.001
	Spinal stenosis at L5/S1	15.9 (3.27–77.7)	<0.001
Deep multifidus fnCSA	**Severity of facet overhang**		
	Moderate facet overhang	0.26 (0.12–0.54)	<0.001
	Severe facet overhang	0.05 (0.03–0.10)	<0.001
	**Male gender**	3.13 (1.94–5.07)	<0.001
	**Multilevel spinal stenosis**	1.30 (0.65–2.60)	0.467
	**Level of spinal stenosis**		
	Spinal stenosis at L3/L4	0.98 (0.43–2.23)	0.968
	Spinal stenosis at L4/L5	0.85 (0.37–1.94)	0.696
	Spinal stenosis at L5/S1	0.41 (0.10–1.67)	0.211

Bridging osteophyte formation was not independently associated with deep part of the multifidus tCSA (*p* = 0.824), fnCSA (*p* = 0.404), or FI (*p* = 0.439) and facet effusion was not independently associated with deep part of the multifidus fnCSA (*p* = 0.319 for small facet effusion; *p* = 0.578 for large facet effusion) or deep part of the multifidus FI (*p* = 0.466 for small facet effusion; *p* = 0.472 for large facet effusion) after controlling for severity of facet overhang.

In subgroup analysis, severe facet overhang was significantly associated with decreased deep part of the multifidus fnCSA in patients <70 years of age (median = 0.5 cm^2^; IQR = 0.2–0.9 cm^2^) and critically decreased deep part of the multifidus fnCSA in patients ≥70 years of age (median = 0.3 cm^2^; IQR = 0.1–0.5 cm^2^).

## 4 DISCUSSION

Extensive effort has been devoted to the study of paraspinal muscle morphology and function, both in healthy individuals and in patients suffering from degenerative conditions of the spine(Cooley et al., 2018; Fortin et al., 2017; Jermy et al., 2020; Wang et al., 2020). However, key pathophysiological mechanisms contributing specifically to deep part of the multifidus atrophy remain poorly understood and the relationship between degenerative facet joint disease and multifidus atrophy has not been rigorously evaluated.

Our results indicate that development of some degenerative changes of the facet joint are correlated with atrophic changes of the multifidus muscle. Moreover, atrophic changes are more prominent in the deep region of the multifidus muscle, where CSA was reduced and increased FI was observed. We have defined facet overhang as a novel parameter, which is strongly associated with deep part of the multifidus atrophy. Our study showed high prevalence of severe facet overhang in patients with lumbar spinal stenosis and in female patients. In contrast, in a previous study by Tamai et al facet joint opening and bridging osteophyte formation were not independently associated with deep part of the multifidus atrophy (Tamai et al., 2017).

Several proposed mechanisms can lead to multifidus muscle atrophy in the setting of degenerative facet changes. Stretching of the joint capsule at its attachment site on the posterolateral margin of the facet joint promotes wrap around bone spur formation (Fujiwara et al., 1999; Varlotta et al., 2011), with potential encroachment into the medial retro‐vertebral space, which is normally occupied by the deep part of the multifidus fibers (Figure 1B). In addition, facet effusion and bridging osteophyte formation are often regarded as indicators of segmental hypermobility(Hasegawa et al., 2010; Kong et al., 2009; Rihn et al., 2007), which may contribute to multifidus denervation from stretching or microtrauma to the medial dorsal ramus (Ozcan‐Eksi et al., 2016). Lastly, high concentrations of proinflammatory mediators have been detected in and are released from facet joint tissues in degenerative spinal disorders (Igarashi et al., 2004; Igarashi et al., 2007; Sugimoto et al., 2019). As such, deep part of the multifidus atrophy may also result from extension of local inflammation (Wang et al., 2021).

Previous studies using CT imaging in a longitudinal population‐based cohort have demonstrated an association between facet joint osteoarthritis and low multifidus density (Kalichman, Hodges, et al., 2010a; Kalichman, Kim, et al., 2010b) as well as higher multifidus fat composition in patients with acute and chronic low back pain(Yu et al., 2017). Facet joint osteoarthritis was defined by qualitative criteria, which included the presence of joint space narrowing, osteophyte formation, hypertrophy of the articular processes, and subarticular bone erosions or subchondral cysts (Weishaupt et al., 1999). However, this grading system suffers, from poor reliability, even in the hands of experienced musculoskeletal radiologists, as evidenced by moderate interobserver agreement for CT and poor to fair interobserver agreement for MR imaging studies (Gellhorn et al., 2013).

Female sex has been previously identified as an independent risk factor for facet arthropathy (Chen et al., 2017; Sniekers et al., 2008), with a dramatic increase in prevalence of facet joint osteoarthritis in females but not in males around the age of menopause (Stromqvist et al., 2016). In addition, sex differences in preoperative clinical assessment scores and an association between higher multifidus atrophy and higher preoperative functional disability in females with lumbar spinal stenosis have recently been reported(Chua et al., 2022; MacLean et al., 2020). Estrogen deficiency was found to accelerate both facet degeneration and multifidus atrophy in females with lumbar spinal stenosis, resulting in a more severe clinical course (Chen et al., 2017; MacLean et al., 2020).

This is a retrospective review of available imaging studies, which were not acquired for all patients using a standardized protocol. Measurement of facet joint angle and identification of bridging osteophyte formation using MR imaging may be more challenging and less accurate. Therefore, in equivocal cases we also reviewed CT imaging, if available. Our study involves patients who underwent surgical decompression for symptomatic lumbar spinal stenosis. This selection bias might affect the generalizability of our findings as these patients suffered from substantial neurogenic claudication that may also have been a contributor to the muscle atrophy beyond the facet joints degeneration. Further studies are needed to examine our finding in asymptomatic patients' cohorts.

## 5 CONCLUSION

We have defined facet overhang as a novel parameter, which is strongly associated with deep part of the multifidus atrophy. In patients with degenerative lumbar spinal stenosis, facet overhang is an independent risk factors for deep part of the multifidus atrophy. Further studies are necessary to explore possible pathological mechanisms that are responsible for this phenomenon and their clinical implications.

## FUNDING INFORMATION

The authors declare that no funds, grants, or other support were received during the preparation of this manuscript.
